# Evaluation of intraventricular flow by multimodality imaging: a review and meta-analysis

**DOI:** 10.1186/s12947-021-00269-8

**Published:** 2021-12-08

**Authors:** Ferit Onur Mutluer, Nikki van der Velde, Jason Voorneveld, Johan G. Bosch, Jolien W. Roos-Hesselink, Rob J. van der Geest, Alexander Hirsch, Annemien van den Bosch

**Affiliations:** 1 Department of Cardiology, Erasmus MC, University Medical Center Rotterdam, P.O. Box 2040, 3000 CA Rotterdam, The Netherlands; 2 Department of Cardiology, Yeditepe University Hospital, Istanbul, Turkey; 3 Department of Radiology and Nuclear Medicine, Erasmus MC, University Medical Center Rotterdam, Rotterdam, The Netherlands; 4 Biomedical Engineering, Department of Cardiology, Erasmus University Medical Center, Rotterdam, The Netherlands; 5 Department of Radiology, Division of Image Processing, Leiden University Medical Center, Leiden, The Netherlands

**Keywords:** Intraventricular flow, Flow mapping, Flow visualization, Multimodality imaging, vortex flow, 4d-flow cardiovascular magnetic resonance, Echocardiography

## Abstract

**Background:**

The aim of this systematic review was to evaluate current inter-modality agreement of noninvasive clinical intraventricular flow (IVF) assessment with 3 emerging imaging modalities: echocardiographic particle image velocimetry (EPIV), vector flow mapping (VFM), and 4-dimensional flow cardiovascular magnetic resonance imaging (4D flow CMR).

**Methods:**

We performed a systematic literature review in the databases EMBASE, Medline OVID and Cochrane Central for identification of studies evaluating left ventricular (LV) flow patterns using one of these flow visualization modalities. Of the 2224 initially retrieved records, 10 EPIV, 23 VFM, and 25 4D flow CMR studies were included in the final analysis.

**Results:**

Vortex parameters were more extensively studied with EPIV, while LV energetics and LV transport mechanics were mainly studied with 4D flow CMR, and LV energy loss and vortex circulation were implemented by VFM studies. Pooled normative values are provided for these parameters. The meta- analysis for the values of two vortex morphology parameters, vortex length and vortex depth, failed to reveal a significant change between heart failure patients and healthy controls.

**Conclusion:**

Agreement between the different modalities studying intraventricular flow is low and different methods of measurement and reporting were used among studies**.** A multimodality framework with a standardized set of flow parameters is necessary for implementation of noninvasive flow visualization in daily clinical practice. The full potential of noninvasive flow visualization in addition to diagnostics could also include guiding medical or interventional treatment.

**Supplementary Information:**

The online version contains supplementary material available at 10.1186/s12947-021-00269-8.

## Background

The assessment of intraventricular flow (IVF) is a potential novel indicator for global ventricular function. Earlier evidence from studies on IVF by Doppler imaging suggested that these complex flow patterns and vortex formation in the left ventricle (LV) have further clinical importance in both physiological and in pathological conditions [1]. The advent of novel imaging modalities as well as advances in software and hardware technology made it possible to study these complex IVF patterns in more detail.

Noninvasive clinical evaluation of IVF and vortex formation became available with the following 3 imaging modalities in the last two decades [2–4] (Table 1):

**Table 1:** Comparison of left ventricle flow visualization modalities

	Echocardiographic particle image velocimetry	Vector flow mapping	4D flow Cardiovascular Magnetic Rensonce
Signal source	Contrast Echocardiography	Color Flow Doppler	Phase contrast MRI
Flow Analysis	2D	2D	3D
Temporal resolution	60 Hz	27–40 Hz	20–40 frames/cycle*
Spatial resolution	3^2^–4^2^ mm^2^	2^2^ mm2	1.5^3^–3^3^ mm3
Upper velocity limitation	40 cm/sec	2 x Nyquist limit	VENC ⁑
Low velocities	Accurate	Underestimation	Accurate†
Scan Time	< 5 min	< 5 min	5–15 min
Commercial hardware	+	+	+
Commercial software	+	+	–
Offline/online	Offline	Offline/Online ‡	Offline
Contrast	Required	Not required	Not required^✽^
Cost	Lower	Lower	Higher
Availability	Higher	Higher	Lower
Dependence on Chest anatomy	+	+	–
Medical implants	§	§	§
Assessment of LV vortex	↑↑↑	↑	↑↑
Assessment of energetics	↑	↑↑	↑↑↑
Volume analysis	–	–	↑↑↑
Estimation of pressure fields	↑↑	↑↑	↑↑
Next generation	• High frame rate echocardiography • Blood speckle tracking • 3D-PIV	• 3D-iVFM • Doppler Vortography	• Multipoint 5D flow CMR

Abbreviations *VENC* velocity encoding, *CHD* congenital heart disease, *3D-PIV* 3-dimensional particle image velocimetry*, 3D-iVFM* 3-dimensional vector flow mapping *number of reconstructed phases, this value is set by the operator ⁑ usually set 150,200 cm/sec. † underestimation of turbulent - nonlaminar flow ‡ the VFM analysis software is integrated on Hitachi echocardiography machines ✽ contrast is not strictly necessary but might increase signal to noise ratio,§. increased artifact with prosthetic material, difficulty in obtaining images in proper angles with assist device cannulae, increased artifact generation with resultant decrease in image quality as well as safety concerns, (−): not applicable/not available (+): present, objective strength in assessments (−) not applicable/not available, ↑: lower strength, ↑↑: moderate strength, ↑↑↑: higher strength

1) Echocardiographic particle image velocimetry (EPIV) tracks the motion of intravenously injected ultrasound contrast agents (UCA) in (usually 2D) ultrasound image sequences to obtain a Eulerian velocity field over the whole region of interest (usually the LV) [5, 6]. Main advantages are bedside applicability, low cost and high spatiotemporal resolution. Main limitations are the necessity of UCA, planar simplification arising from the assumption that the speckles stay in-plane, and dependence on chest anatomy [7].

2) Vector flow mapping (VFM) utilizes color Doppler imaging (CDI) for constructing velocity fields [2, 8]. Frame-by-frame analysis reveals flow patterns in the LV [9]. It allows bedside evaluation of IVF without the need of UCA. There are several limitations of this technique. Because frame rate could drop below 20 Hz when imaging the LV, data from multiple cardiac cycles are combined to improve the velocity tracking accuracy. In addition, calculation of angular component of the velocity vectors is based on various different algorithms in different approaches and none of these algorithms are validated thoroughly [7].

3) 4-dimensional flow cardiovascular magnetic resonance imaging (4D flow CMR) is a technique with time-resolved flow-encoding capabilities in three spatial dimensions. 3D velocity encoding resulted in the evolution of 4D flow CMR from the conventional 2D-phase contrast CMR (2D-PC CMR) (10). The main advantage of this technique is the 3D flow assessment and independence of the chest anatomy, while the main disadvantages would be longer scan times, limited accuracy in presence of turbulent flows, relatively low availability and higher cost compared with ultrasound based modalities (11) (Table 1).

Despite the well-established framework of IVF with each modality separately, the inter-modality agreement with regard to quantitative measures of normal IVF was not studied before. Therefore, we aimed in this current review 1) to evaluate inter-modality implementation of parameters of IVF, 2) to provide normative values for parameters that are frequently reported in the literature, 3) to perform a meta-analysis for changes in flow parameters in patients with LV systolic dysfunction, and 4) to review the future perspectives in the field of noninvasive IVF imaging.

## Methods

### Search strategy

A systematic review was conducted for evaluating inter-modality agreement in analysis of LV flow. The search was performed in compliance with Preferred Reporting Items for Systematic Reviews and Meta-Analyses (PRISMA) statement (12). The search was performed on February 11th, 2019 in the databases EMBASE, Medline OVID, and Cochrane central with structured keywords (Supplementary Table 1). Following deduplication, two independent authors (FOM, AvdB) performed title and abstract screening (13).

### Inclusion and exclusion criteria

The current study focused on the IVF findings in healthy adults. Studies that enrolled at least 10 healthy participants and examined LV flow patterns with EPIV, VFM, or 4D flow CMR were included. Studies conducted on animals, children, or in-vitro models were excluded. Additionally, studies published in a language other than English were also excluded. Supplementary Tables 2, 3, and 4 display the list of the included studies for EPIV, VFM, and 4D flow CMR, respectively.

### Data extraction

One author (FOM) extracted all the relevant data using a spreadsheet. A second author verified these findings (AvdB). The following data were collected: year of publication, first author, number of healthy participants / patients, quantitative measures, and qualitative descriptors of IVF.

### Data analysis

Continuous variables were presented as mean ± standard deviation. Categorical variables were presented as percentages. Normative values of parameters implemented by multiple studies were calculated, when the method of measurement was uniform. A meta-analysis procedure was executed for two parameters reported both in healthy adult controls and heart failure patients: vortex depth and vortex length. Heterogeneity indexes (*I*^*2*^) were calculated for the pooled results with the metaphor package, in R statistical software (version 3.5.2) (14). The random effects model was utilized for the calculation of the heterogeneity index.

## Results

**Fig. 1:**
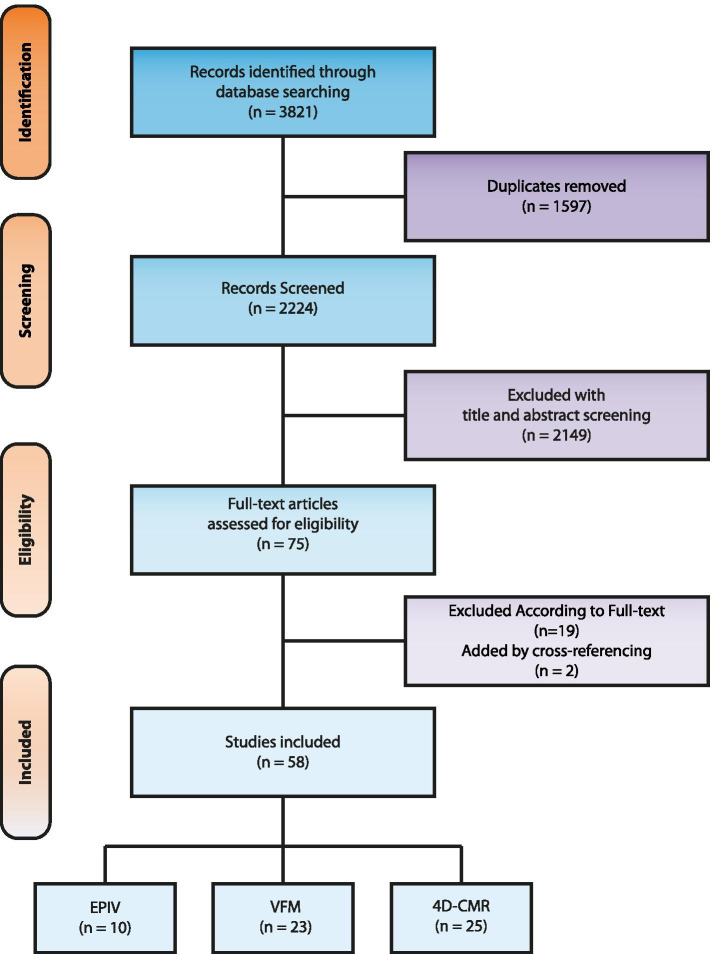
Inclusion of studies, flowchart modified from the Prisma statement for reporting systematic reviews and meta-analyses [12]

Figure 1 displays the PRISMA chart of the study inclusion process. Two studies were found and added by cross-referencing (15, 16). Of the finally included 58 studies, 10 utilized EPIV, 23 VFM, and 25 4D flow CMR.

The IVF parameters were classified in four major groups: parameters of 1) LV vortex, 2) LV energetics, 3) LV transport mechanics, 4) LV pressure and velocity fields. A previous framework suggested by Mele et al. was modified for this classification (5). While EPIV studies paid more attention to LV vortex analysis, 4D flow CMR studies more often focused on characterizing LV energetics and LV transport mechanics. VFM studies also mainly focused on LV energetics. There were four 4D flow CMR, three EPIV and two VFM studies that reported intraventricular velocity and pressure profiles. These parameters are summarized in Supplementary Table 5.

**Fig. 2:**
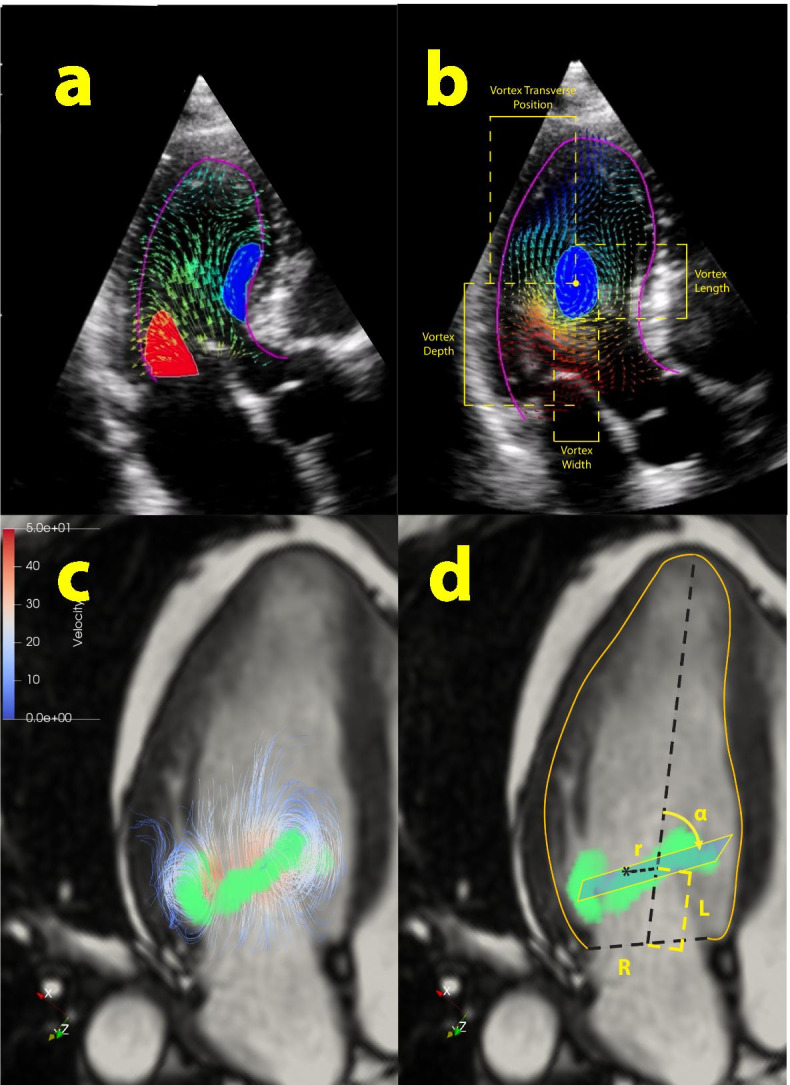
A. Early diastolic vortex ring demonstrated by EPIV, with clockwise anterior (blue) and anti-clockwise posterior (red) arms. B. 2-dimensional quantification of the late diastolic vortex core by Hyperflow software. Vortex length and vortex depth are indexed to LV apicobasal length while vortex width and vortex transversal position are indexed to LV posterioseptal length. C. Three dimensional structure of the toroid early diastolic LV vortex demonstrated by 4D flow CMR D. 3-dimensional quantification of the early diastolic vortex ring. Radial coordinate (r) is indexed to basal endocardial radius (R). Longitudinal coordinate (L) is indexed to LV apicobasal length. Vortex ring orientation (α) is the angle between the vortex plane and the LV longitudinal axis

Table 2 demonstrates LV vortex parameters. These parameters defined four aspects of LV vortex: 1) size and localization, 2) timing, 3) extent of vortical (or swirling) motion, and 4) the extent of fluctuations in LV vortex throughout the cardiac cycle. Vortex area/or volume was reported by all three modalities. However, there was no inter-modality standardization on methods of measurement for this parameter. While the ultrasound-based modalities have reported 2D vortex parameters, 4D flow CMR allowed the 3D characterization of the torus-shaped LV vortex (Fig. 2) (17). Vortex circulation, although terming varied among studies, was another LV parameter implemented by multimodality imaging. There was significant inter-modality heterogeneity with regard to value ranges (15).

**Table 2:** Left ventricular vortex parameters by multimodality imaging

Parameters	Definition	EPIV	VFM	4D flow CMR	Clinical implications	Ref.
**Size and localization of the vortex**					Increased vortex depth, length, radius and area values in patients with LV systolic dysfunction	[s.1–7]
*Vortex length*	Apico-basal diameter indexed to LV length	+	–	+		
*Vortex width*	Mediolateral diameter indexed to LV transverse width	+	–	+		
*Vortex radius*	Maximum or mean radius/diameter of the vortex	–	+	–		
*Vortex size*	Vortex area, absolute value or indexed to LV area	**+**	**+**	**+**		
*Vortex depth*	Distance to the mitral annulus indexed to LV length	+	–	+		
*Vortex transversal position*	Distance of the vortex core to septum, indexed to LV length	+	–	+		
*Vortex sphericity index*	Vortex width indexed to vortex length	+	–	+		
**Timing and duration of the vortex**					Delayed diastolic vortex formation, longer duration of persistence of vortex during ejection time despite unchanged total vortex duration in DCM patients	[s.6, 8–10]
*Vortex duration*	Duration of vortex in the LV	+	+	–		
*Time to diastolic vortex formation*	Time from mitral valve opening to vortex formation	+	–	–		
**Vorticity parameters**					Significant heterogeneity in reported value ranges as well as the direction of change with diseases compared with controls	[s.3, 6, 8, 11–21]
*Vortex circulation*	Vorticity over the main diastolic vortex	**+**	**+**	**+**		
*Vortex strength*	Clockwise and counterclockwise vorticity indexed to total LV circulation	+	–	–		
*Vorticity fluctuation index*	Standard deviation of the squared vorticity	+	–	–		
*Reynolds number*	V.D/v, where V is linear velocity, D is the diameter of flow chamber and v is the kinematic viscosity. Higher number indicates increased vortical flow.	–	+	+	Higher mean Reynolds numbers in the LV in patients with heart failure. No difference between controls and patients with Fontan circulation.	[s.8, 21]
**Vortex pulsatility parameters**						[s.16, 22, 23]
*Relative strength*	Pulsatile/nonpulsatile vorticity over LV	+	–	–	Lower in DCM patients, higher in patients with left bundle branch block compared to healthy controls.	
*Vortex relative strength*	Pulsatile/nonpulsatile vorticity over vortex	+	–	–		
*Vortex Pulsation correlation*	Correlation of steady and pulsatile vortex components with zeroeth order vorticity	+	–	–		

*EPIV* echocardiographic particle image velocimetry, *VFM* vector flow mapping, *4D flow CMR* 4-dimensional flow cardiovascular magnetic resonance, *LV* left ventricle, *DCM* dilated cardiomyopathy, (−) not available, (+) available

Parameters of LV energetics and LV blood transport mechanics are demonstrated in Table 3. All three modalities reported energy expenditure (or loss) over the LV; however, the measurement methods differed. A unitless cumulative kinetic energy dissipation index was reported by EPIV whereas a viscous planar energy loss value was reported by VFM. 4D flow CMR was superior in the fact that three different parameters of energetics; viscous energy loss, kinetic energy dissipation, and turbulent kinetic energy could be reported in a 3D manner. Parameters of LV blood transport mechanics were mainly studied with the use of 4D flow CMR. One of these parameters, Vdirect, is the volume that enters and leaves LV in a single beat, and has been suggested as a measure of LV pump efficiency and found to be decreased in patients with impaired LV systolic function. There is low level of agreement between modalities with regard to parameters of the IV velocity and pressure profiles. These parameters are presented in Supplementary Table 5.

**Table 3:** Parameters of left ventricular energetics / blood transport mechanics

Parameter	Definition	EPIV	VFM	4D flow CMR	Clinical Implications	Ref.
**LV Energetics**						[s.11, 21, 24–32]
*Kinetic Energy Dissipation*	Kinetic energy over LV	+	–	+	Kinetic energy dissipation was increased in post-MI patients with LVEF > 50%, decreased in patients with ischemic LVSD and TOF, increased in patients with DCM and Fontan circulation. Turbulent kinetic energy was increased in DCM patients, the values increase with increasing size of LV. Energy loss was increased with cardiovascular disease/systemic diseases with cardiovascular involvement.	
*Turbulent kinetic energy*	Velocity fluctuation intensity in perpendicular directions	–	–	+		
*Energy loss*	Total energy loss dissipated as kinetic energy and viscous friction	–	+	+		
**LV blood transport mechanics**						[s.11, 33]
Direct volume	Volume of blood entering the LV and leaving in the analyzed beat	+	–	+	Vdirect was the parameter with the most robust evidence. It has shown to be a surrogate marker of LV energetic efficiency. It constitutes the majority of the VEDV, maintains a position closer to LVOT and a smaller angle to LVOT axis and correlates with LVEF significantly.	
Delayed ejection volume	Volume not entered the LV in the previous beat but ejects in the analyzed beat	–	–	+		
Retained flow	Volume entering the LV but not ejecting in the analyzed beat	–	–	+		
Residual flow	Volume not entering and not leaving in the analyzed beat	–	–	+		

EPIV: echocardiographic particle image velocimetry, VFM: vector flow mapping, 4D flow CMR: 4-dimensional flow cardiovascular magnetic resonance imaging, LV: left ventricle, LVSD: left ventricle systolic dysfunction, TOF: Tetralogy of Fallot, DCM: dilated cardiomyopathy, MI: myocardial infarction, LVEF: left ventricle ejection fraction, LVEDV: left ventricle end-diastolic volume, LVOT: left ventricle outflow tract, (−) not available, (+) available

**Fig. 3:**
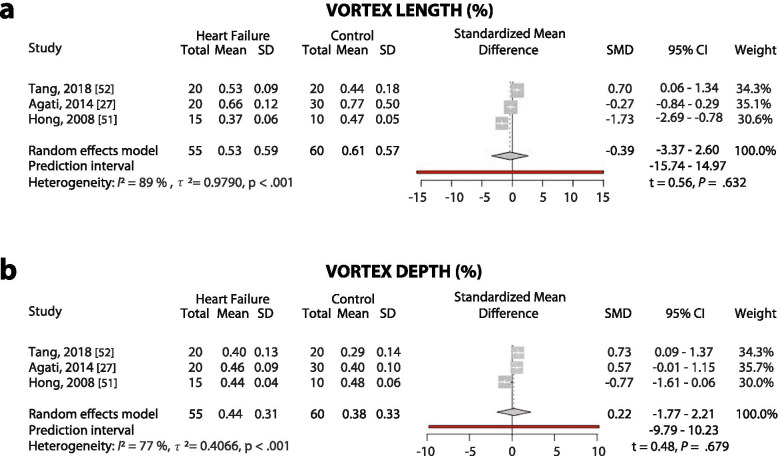
Meta-analysis for vortex length (A) and depth (B) in heart failure with reduced ejection fraction patients versus healthy controls with EPIV. SD: standard deviation, SMD: standard mean difference, CI: confidence interval

Normative values of the commonly reported LV flow parameters are summarized in Table 4. The most commonly reported parameters (in more than 100 patients) in healthy individuals were energy loss, vortex circulation, vortex length, and vortex area. A meta-analysis was performed for change of LV flow in patients with LV systolic dysfunction for two vortex parameters: vortex length and vortex depth (Fig. 3). A high heterogeneity index with no significant difference between controls versus heart failure with reduced ejection fraction patients was demonstrated for both parameters (Vortex Length: 0.53 ± 0.59 versus 0.61 ± 0.57, t = 0.56, *p* = .632, I^2^ = 89%, *p* < .001; Vortex Depth: 0.44 ± 0.31 versus 0.38 ± 0.33, t = 0.48, *p* = .679, I^2^ = 77%, p < .001, for heart failure patients versus healthy adults, respectively).

**Table 4:** Normative ranges for the parameters of intraventricular flow

	Imaging modality	Pooled n	Mean ± S.D.	Ref.
Size and localization of the vortex				
Vortex Area (/LVEDA)	EPIV	104	0.29 ± 0.08	[s.10, 11, 13, 15, 22, 24]
Vortex length (/LV width)	EPIV	126	0.61 ± 0.27	[s.10, 11, 13, 16, 22–24, 34]
Vortex depth (/LV width)	EPIV	85	0.40 ± 0.11	[s.11, 16, 22–24]
Vorticity Parameters				
Vortex Circulation (cm2/s)	VFM	135	16.3 ± 11.1	[s.3, 6, 19]
Vortex Pulsatility Parameters				
Relative Strength	EPIV	35	1.6 ± 0.6	[s.16, 22, 23]
Vortex Relative Strength	EPIV	35	0.6 ± 0.3	[s.16, 22, 23]
Vortex Pulsation Correlation	EPIV	35	0.3 ± 0.6	[s.16, 22, 23]
LV Energetics				
Kinetic Energy Dissipation	EPIV	80	0.5 ± 0.2	[s.11, 13, 24, 34]
Kinetic Energy Fluctuation	EPIV	60	1.8 ± 0.3	[s.11, 13, 24]
Energy Loss Systolic (mW/m)	VFM	203	16.2 ± 7.2	[s.29, 30, 35, 36]
Energy Loss Diastolic (mW/m)	VFM	203	22.7 ± 8.2	[s.29, 30, 35, 36]
Peak kinetic energy systole (mJ)	4D flowCMR	44	3.9 ± 1.1	[s.21, 37, 38]
LV blood transport mechanics				
Vdirect (%)	4D flow CMR	74	37 ± 5	[s.33, 39–41]
Retained flow (%)	4D flow CMR	74	18 ± 5	[s.33, 39–41]
Delayed ejection flow (%)	4D flow CMR	74	17 ± 3	[s.33, 39–41]
Residual volume (%)	4D flow CMR	74	28 ± 5	[s.33, 39–41]

*E-PIV* echocardiographic particle image velocimetry, *VFM* vector flow mapping, *4D flowCMR* 4-dimensional flow cardiovascular magnetic resonance, *LVEDA* left ventricle end-diastolic area, *EDV* end-diastolic volume

## Discussion

Identification of flow patterns in the heart and their clinical implications has evolved as a major endeavor of research in cardiovascular imaging. All the three dedicated noninvasive cardiovascular flow imaging modalities discussed in this paper, are commercially available for clinical use; however, the flow imaging data is still not implemented in clinical practice. The current study demonstrated important pitfalls and areas that need improvement.

The findings from this review could be summarized as follows: 1) there is virtually no inter-modality standardization with regard to methods of analysis, units of measurement or ranges of normative values for IVF parameters, 2) vortex area and vortex length with EPIV, energy loss with VFM; and LV-volumes analyzed by particle tracing with 4D flow CMR were the main parameters over which intra-modality agreement was found, and 3) meta-analysis of the differences of two vortex parameters, vortex length and vortex depth, demonstrated no significant difference between controls and heart failure patients.

Previous validation studies demonstrated accurate evaluation of flow with EPIV (18), VFM (19) and 4D flow CMR (20), in describing velocity fields, mainly against phantom models. Inter-modality agreement of the velocity fields was also evaluated in healthy individuals for VFM versus 4D flow CMR previously (21). However, cross-validation of the parameters calculated by post-processing of the velocity fields, summarized in Tables 2 and 3 is largely lacking, mainly because these parameters are not available on the analysis software of each modality. There is intra-modality agreement with regard to utilized software for EPIV and VFM. For 4D flow CMR, use of in house custom software allows calculation of additional parameters, such as 3D vorticity (22) and 3D morphology parameters (17) (Supplementary Tables 2 and 3, Fig. 2). Additionally, inherent technical limitations of the modalities may result in inaccuracies in measurement of certain parameters with certain modalities. For example, EPIV highly underestimates the velocity value and is inappropriate for KE that is dominated by high velocities; KE dissipation is a combination of velocity gradients and it necessarily depends on the spatial resolution that is very different among modalities; and vortex geometry depends on the pattern of low velocity that is inaccurate in VFM.

Our findings highlight the need for validating of flow parameters used in the studies against gold standard tests and in between modalities, as well as standardization in measurement methods and reporting. We additionally presented normative values for flow parameters over which intra-modality agreement was present. Energy loss, analyzed by VFM, was the most commonly reported parameter and was found increased in cardiovascular disease as well as cardiovascular involvement in systemic disease, such as diabetes mellitus [23] and end-stage renal disease [24]. This parameter was also implemented by 4D flow CMR with the use of experimental software, and was associated with disturbed vortex pattern in patients with surgically repaired atrioventricular septal defect [17].

Parameters of vortex size and localization were comprehensively studied with EPIV. One of them, vortex area, was also implemented by VFM [25] and 4D flow CMR [26]. The finding of larger vortices that descend deeper into the LV in patients with left ventricle systolic dysfunction was confirmed with VFM [25]. In contrast, in our meta-analysis we didn’t observe a statistically significant difference between controls and HF patients, when vortex size is evaluated by vortex length. This shows that the findings from the small observational studies should be interpreted with caution until the changes in parameters in patients are validated in large cohorts. Of note, most clinical studies about LV flow are aimed to search which parameters, if any, may provide useful indications regarding LV function that is incremental with respect to existing parameters. Probably, none of them has been successful so far. Thus, the heterogeneity of results with the different modalities, occasionally also within a same modality, is partly imputable to the fact that these are research-oriented studies, which favored the exploration of potential measures rather than the rigor of their reproducibility.

The LV transport mechanics were studied with delineation of the LV volume fractions by particle tracing with 4D flow CMR. 4D flow CMR findings that this parameter reflects the efficiency of LV as a pump and was decreased in patients with LVSD. A 2D implementation of Vdirect with EPIV demonstrated findings similar to 4D-CMR version. [27]. The role of the Vdirect as a marker of the efficiency of LV transport should be further investigated.

We suggest implementing a small set of 2D parameters that could be measured with all these three modalities. This small set of parameters could be integrated in the flow analysis software of all three modalities, standardized with respect to measurement method and reporting, and should be tested in large cohorts of healthy individuals to determine the reference ranges. Additionally, the modalities should be cross-validated against each other. In this manner, a common terminology and language, which is vital for use in clinical settings, could be reached. There is a clear need for a multimodality flow visualization taskforce, with clinicians and engineers working with different modalities including industry representatives for inter-modality standardization of parameters and to coordinate clinical implementation.

### Future perspectives

Although the novel flow visualization modalities allowed measurement of new parameters, there is no clinical implementation of these parameters. The flow transit (Vdirect) is one of the most promising parameters, although it is still unclear if it provides additional information with respect to EF; energetic properties require a standardization to make them independent of image resolution and acquisition/filtering settings; intraventricular pressure gradient (or hemodynamic force) is another candidate although first results (EPIV and 4D flow CMR) are not yet conclusive. Inherent technical limitations causing decreased accuracy is probably the most important limiting factor in implementation of these novel parameters in the clinical practice.

There is ongoing research that aims overcoming the limitations of the current modalities. High frame rate echocardiography is an upgrade to EPIV which was shown to accurately track IVF velocities [28]. For pediatric imaging, blood speckle tracking using high frame rate echocardiography, can estimate blood velocity fields without the need for ultrasound contrast agent - making it a promising tool for studying congenital heart dysfunction; however its use in adults is currently limited due to low signal to noise ratio at depths greater than 8 cm [29].

There are 2 successors of VFM: Doppler vortography and 3D-iVFM. Doppler vortography relies on a fast detection algorithm for identification of vortex cores on CDI acquisition [30]. This technique was recently combined with high frame rate imaging and demonstrated good to excellent agreement with 4D flow CMR in measurement of LV vorticity [31]. 3D-iVFM, based on 3D ultrafast Doppler imaging, allowed temporal resolutions more than 1000 volumes/sec with promising initial in-vivo results [32]. The 3-dimensional EPIV and VFM might allow the same 3D parameters as 4D flow CMR to be measured.

For 4D flow CMR, intravoxel velocity standard deviation mapping, or turbulence mapping is based on introduction of additional velocity encoding points for each spatial axis. With Bayesian processing, this method allows the turbulent flow in voxels to be estimated. Respiratory motion resolved imaging instead of respiratory gating is introduced to make scan times shorter and more predictable. The combination of these 2 techniques is termed Multipoint 5D Flow MRI [33]. Automatic and semi-automatic segmentation decrease analysis time, intra and interobserver variability and as a result is expected to increase precision in the future [34].

## Conclusions

The inter-modality agreement with regard to intracardiac flow parameters is not sufficient for clinical implementation, a single parameter that is measured the same way with all 3 modalities is missing. A standardized set of flow parameters, with well-defined reference ranges will substantially facilitate translation of the insights from IVF visualization to clinical practice. Coordinated interdisciplinary efforts of professionals working in the field, along with technical advances will unleash the full potential of flow visualization in diagnosis and treatment of cardiovascular disease.

## Supplementary Information

**Additional file 1:** **Supplementary table 1.** Keywords used for database search. **Supplementary table 2.** Included EPIV studies. **Supplementary table 3.** Included and excluded VFM studies. **Supplementary table 4.** Included and excluded 4D flow CMR studies. **Supplementary table 5.** Intraventricular velocity and pressure profiles.

**Additional file 2.** Supplementary references

**Additional file 3.** Graphical abstract. Noninvasive flow visualization with multimodality imaging.

## Data Availability

The datasets used and/or analysed during the current study are available from the corresponding author on reasonable request.

## Abbreviations

IVFf: Intraventricular flow
EPIV: Echocardiographic particle image velocimetry
VFM: Vector flow mapping
4D flow CMR: 4-dimensional flow cardiovascular magnetic resonance imaging
LV: Left ventricle
UCA: Ultrasound contrast agent
CDI: Color doppler imaging
2D-PC CMR: 2D-phase contrast CMR
