# Supplementary material for: Evaluation of intraventricular flow by multimodality imaging: a review and meta-analysis
Source: Cardiovasc Ultrasound. 2021 Dec 8;19:38. doi: 10.1186/s12947-021-00269-8 (PMC8653587; doi:10.1186/s12947-021-00269-8)
Supplement: Supplementary file 1 — Additional file 1: Supplementary table 1. Keywords used for database search. Supplementary table 2. Included EPIV studies. Supplementary table 3. Included and excluded VFM studies. Supplementary table 4. Included and excluded 4D flow CMR studies. Supplementary table 5. Intraventricular velocity and pressure profiles. [file 12947_2021_269_MOESM1_ESM.docx]

## ADDITIONAL FILE 1 - Supplementary tables

| **Supplementary table 1.** Keywords used for database search | |
| --- | --- |
| Embase | *('heart left ventricle'/exp OR 'heart left ventricle ejection fraction'/de OR 'heart left ventricle function'/de OR 'heart left ventricle compliance'/de OR 'heart left ventricle contractility'/de OR 'heart left ventricle contraction'/de OR 'heart left ventricle ejection fraction'/de OR 'heart left ventricle ejection time'/de OR 'heart left ventricle enddiastolic pressure'/de OR 'heart left ventricle enddiastolic volume'/de OR 'heart left ventricle endsystolic volume'/de OR 'heart left ventricle failure'/de OR 'heart left ventricle filling'/de OR 'heart left ventricle filling pressure'/de OR 'heart left ventricle function'/de OR 'heart left ventricle hypertrophy'/de OR 'heart left ventricle infarction'/de OR 'heart left ventricle mass'/de OR 'heart left ventricle muscle'/de OR 'heart left ventricle outflow tract'/de OR 'heart left ventricle outflow tract obstruction'/de OR 'heart left ventricle overload'/de OR 'heart left ventricle performance'/de OR 'heart left ventricle pressure'/de OR 'heart left ventricle relaxation'/de OR 'heart left ventricle size'/de OR 'heart left ventricle volume'/de OR 'heart left ventricle wall'/de OR 'heart left ventricle wall motion'/de OR 'heart left ventricle work'/de OR ((left* NEAR/3 (ventric* OR intraventric*) NOT (lvad OR 'assist device'))):ab,ti,de) AND (('dynamics'/de AND 'blood flow'/de) OR 'vortex motion'/de OR 'computational fluid dynamics'/exp OR 'blood flowmetry'/de OR (((flow OR fluid) NEAR/3 (dynamic* OR analys* OR pattern* OR mechanic* OR mapping OR parameter* OR measurement* OR quantificat* OR imaging)) OR vortex OR vortices OR fluidynamic* OR biofluidynamic* OR velocimet*):ab,ti) AND ('nuclear magnetic resonance imaging'/exp OR 'echography'/de OR 'echocardiography'/exp OR 'color Doppler flowmetry'/de OR ((magnetic* NEAR/3 resonan*) OR mri OR echogra* OR echocardiogra* OR Doppler* OR mr OR ultraso*):ab,ti) NOT ([animals]/lim NOT [humans]/lim) NOT ([Conference Abstract]/lim) AND [english]/lim* |
| Medline Ovid | *(((left* ADJ3 (ventric* OR intraventric*) NOT (lvad OR assist device))).ab,ti.) AND ((((flow OR fluid) ADJ3 (dynamic* OR analys* OR pattern* OR mechanic* OR mapping OR parameter* OR measurement* OR quantificat* OR imaging)) OR vortex OR vortices OR fluidynamic* OR biofluidynamic* OR velocimet*).ab,ti.) AND (exp Magnetic Resonance Imaging/ OR Ultrasonography/ OR exp Echocardiography/ OR Laser-Doppler Flowmetry/ OR ((magnetic* ADJ3 resonan*) OR mri OR echogra* OR echocardiogra* OR Doppler* OR mr OR ultraso*).ab,ti.) NOT (exp animals/ NOT humans/) AND english.la.* |
| Cochrane Central | *(((left* NEAR/3 (ventric* OR intraventric*) NOT (lvad OR 'assist device'))):ab,ti) AND ((((flow OR fluid) NEAR/3 (dynamic* OR analys* OR pattern* OR mechanic* OR mapping OR parameter* OR measurement* OR quantificat* OR imaging)) OR vortex OR vortices OR fluidynamic* OR biofluidynamic* OR velocimet*):ab,ti) AND (((magnetic* NEAR/3 resonan*) OR mri OR echogra* OR echocardiogra* OR Doppler* OR mr OR ultraso*):ab,ti)* |

| **Supplementary table 2.** Included EPIV studies | | | | | | | | |
| --- | --- | --- | --- | --- | --- | --- | --- | --- |
| Study, year | | UCA | Hardware platform | Software Platform | Frame rate (Hz) | Healthy controls (n) | Patient Group (n) | Notes/clinical implications |
| Abe, 2013 [s.12] | | Definity* | Vivid 7 | Hyperflow† | 204 ± 39 | 19 | HF with Preserved (10) and Reduced (13) ejection fraction | Vortex Strength during isovolumic contraction (VS_IC_) decreased in patients with HFPEF and HFREF compared to controls. |
| Agati, 2014 [s.11] | | Sonovue‡ | Philips IE33 | Hyperflow | 80 | 30 | Post-MI patients LVEF > 50 % (14)  LVEF 30 – 50 % (10)  LVEF < 30 % (10) | Energy dissipation, energy fluctuation, vorticity fluctuation and direct volume fraction (V_direct_) decreaed with decreasing LVEF. |
| Cimino, 2012* [s.13] | | Sonovue | N/A | Hyperflow | 80 | 10 | N/A | Cited in [s.11]. Values of vortex morphology, vorticity and vorticity fluctuation, kinetic energy dissipation and kinetic energy dissipation values reported in the healthy individuals. |
| Goliasch, 2013 [s.10] | | Sonovue | Vivid 7 | Hyperflow | N/A | 11 | CRT patients (11) | VL, VA and vortex intensity are greater in controls compared with patients with CRT. Diastolic vortex formation is delayed in HF patients and becomes more delayed when CRT is deactivated. |
| Gurel, 2016 [s.22] | | Sonovue | BT 09 | Q flow§ | 95-110 | 10 | AAI pacing (20) DDD pacing (20)  LBBB (6) | VA, VL, RS, VRS, VPC smaller in AAI and normal subjects compared to DDD and LBBB. VD larger in Normal subjects, patients with LBBB and DDD compared with patients with AAI. Later onset of Vortex formation and shorter vortex duration in LBBB and DDD patients compared to AAI and NSR |
| Hong, 2008 [s.23] | | Definity | Sequoia C512 | Omegaflow | 60 - 80 | 10 | DCM (15) | VL, VW and VD higher in controls |
| Kutty, 2014 [s.15] | | Definity | Sequoia C512 | Hyperflow | 60 | 23 | TOF (14) | VA lower and kinetic energy dissipation higher in controls compared to patients with TOF |
| Lampropoulous, 2012 [s.16] | | Sonovue | Sequoia C512 | Omega Flow\| | N/A | 15 | Patients with Fontan circulation (8) | VL and SI higher, VW and VD lower in healthy controls compared to Fontan patients |
| Prinz, 2014 [s.34] | | Sonovue | GE Vingmed | Hyperflow | 100-120 | 20 | HNCMP with LVDD grade 1 (13), grade 2 (12) and grade 3 (5) | VL, VW and SI as well as pressure and velocity gradients between mid-LV and LA higher, energy dissipation significantly lower im healthy controls compared with HCM patients with diastolic dysfunction |
| Tang, 2018 [s.24] | | not mentioned | LOGIQ E9 | Hyperflow | N/A | 20 | DCM (20) | VA, VL, VD, KED higher in DCM. Mean LV flow vector is more horizontal in patients with DCM. |
|  | EPIV = echocardiographic particle image velocimetry, UCA = ultrasound contrast agent, HF = heart failure, N/A = not available, CRT = cardiac resynchronization therapy, VA = vortex area, VL = vortex length, VW = vortex width, RS = relative strength, VRS = vortex relative strength, VPC = vortex pulsation correlation, SI = sphericity index, VD = vortex depth, LBBB = left bundle branch block, DCM = dilated cardiomyopathy, TOF = tetralogy of Fallot, HNCMP = hypertrophic nonobstructive cardiomyopathy, LVDD= left ventricle diastolic dysfunction, LV = left ventricle, EF = ejection fraction, MI = myocardial infarction, *Lantheus Medical Imaging, Inc., North Billerica, MA, USA, †AMID, Trieste, Italy, ‡Bracco, Milan, Italy, §Siemens Healthcare, Erlangen, Germany, \|Siemens, Mountain View, USA ● included manually | | | | | | | |

| **Supplementary table 3.** Included VFM studies | | | | | |
| --- | --- | --- | --- | --- | --- |
| Study, year | Software Platform | Frame Rate (Hz) | Healthy controls (n) | Patient Group (n) | Notes/clinical implications |
| Akiyama, 2017 [s.36] | DAS-RS1* | N/A | 50 | None | LV EL, KE in LVOT and KEcycle/ELcyclewere reported healthy adults. |
| Bermejo, 2014 [s.8] | Custom software | 30 ± 8 | 61 | DCM (61) | Larger and stronger vortices in DCM compared with controls. |
| Chen, 2012 [s.9] | DAS-RS1 | N/A | 30 | Uremia (33) | Size and duration of vortex during ejection were higher in uremic patients compared with controls. |
| Chen, 2013 [s.42] | DAS-RS1 | ≥ 30 | 22 | Impaired relaxation (34), restrictive filling (27) | Intraventricular apicobasal velocity gradient was higher in controls compared to patients with diastolic dysfunction. |
| Fukuda, 2014 [s.43] | DAS-RS1 | 45 ± 5.3 | 20 | DCM  nonischemic (29) ischemic (31) | Vortex duration/ejection time was higher in patients with ischemic LVSD compared with controls |
| Ji, 2018 [s.28] | DAS-RS1 | +20 | 40 | HCM (40) | EL during IVC was higher in HCM patients, EL during early diastole was higher in controls. |
| Li, 2017 [s.29] | DAS-RS-1 | 20 - 25 | 58 | DM  controlled (58)  uncontrolled (58) | Diastolic EL > Systolic EL in healthy controls. EL was higher in controlled DM and highest in uncontrolled DM patients compared to controls. |
| Li, 2019 [s.3] | DAS-RS-1 | 53 ± 9 | 36 | ischemic DCM (76)  CAD (61) | Vortex resided in basal segments and LVOT velocities were higher in controls. Apical localization of vortices in patients with LVSD. |
| Lin, 2018 [s.30] | DAS-RS-1 | 22 | 44 | AF (53) | ELsys/ELed ratio was higher in patients with AF compared to controls. This disturbance in energetic efficiency recovered during short-term follow-up after RFCA. |
| Martinez, 2014 [s.17] | Custom software | N/A | 20 | NIDCM (20)  HCM (20) | Contribution of vortical flow to LV filling increased in patients with NIDCM and decreased in patients with HCM |
| Munoz, 2015 [s.18] | DAS-RS-1 | N/A | 48 | N/A | Relative duration of late diastolic vortex was shorter in healthy adults with impaired relaxation (e’< 8 cm/s) |
| Nogami, 2013 [s.44] | DAS-RS-1 | 30 ± 8 | 36 | Impaired relaxation (25) | Time to peak KE during diastole longer in mid and apical LV higher in patients with pseudonormal relaxation and low LVEF was longer. Basal LV energy loss values higher compared with mid and apical values in patients and controls. |
| Nogami, 2014 [s.45] | DAS-RS-1 | 30 ± 8 | 36 | HF (24) | Presence of an EL peak before E peak, termed suction flow, is present in all controls but half of the patients with elevated filling pressures. LV twist was lower in patients with absent suction pattern. |
| Ro, 2014 [s.19] | DAS-RS-1 | 38,5 ± 10 | 37 | HCM  nonobstructive (23)  obstructive (22) | Angle of velocity vectors to mitral leaflets higher in HOCM patients, ricochet vectors, absent in controls and more frequent in HOCM compared with HNCM. |
| Wang, 2016 [s.31] | DAS-RS-1 | +60 | 38 | Prediabetes (30) Diabetes (51) | EL values were increased in prediabetes and diabetes patients compared with controls. |
| Xu, 2017 [s.35] | DAS-RS-1 | 19-28 | 51 | N/A | EL range in healthy adults was reported. EL correlated with AVTI, A wave peak velocity and IVCT |
| Yoshida, 2019 [s.5] | DAS-RS-1 | 27-40 | 16 | Repaired MV (52)  Normal (33)  Depressed EF (19)  Prosthetic MV (37)  Normal (19)  Depressed EF (18) | EL is greater in repaired and greatest in prosthetic mitral valves. EL in all patient groups lower when LVEF is lower. |
| Zhang, 2012 [s.46] | DAS-RS1 | 63±5  vs  45±5 | 58 | HF (61) | Vortex area decreased significantly during IVC in HF patients but didn’t change in controls. Vortex circulation decreased during IVC in all participants. |
| Zhang, 2016 [s.47] | DAS-RS1 |  | 41 | CAD (29)  DCM (41) | Increasing level of vortical flow during isovolumic relaxation was found associated with impaired LV systolic and diastolic function |
| Zhang, 2013 [s.6] | DAS-RS1 | 52 ± 9 | 62 | CAD  Normal EF (51)  Depressed EF (70) | Vortex duration and vortex duration/ ejection time were greater in patients with low EF. % VA and area flux change from start of ejection to peak ejection was smaller in patients with low EF compared with controls. |
| Zhong, 2016 [s.32] | DAS-RS-1 | 20-25 | 50 | Patients preparing for Hemodialysis (21), on hemodialysis (21), on periton dialysis (21) | Systolic and diastolic EL were higher in patients with ESRD. |
| Zhou, 2015 [s.48] | DAS-RS-1 | N/A | 38 | Simple HT (37) | More vortical flow observed in HT patients. Peak systolic velocity and flow, velocity gradient apex to aortic valve were higher in HT compared to controls. |
| Zhou, 2017 [s.7] | DAS-RS-1 | ≥ 25 | 27 | Simple HT (21),  Thyrotoxic cardiomyopathy (22) | Circulation and intraventricular pressure gradient were higher in simple HT compared to thyrotoxic CMP. VA was greater in thyrotoxic CM and greatest in simple HT. |
| VFM = vector flow mapping, N/A = not available, LV = left ventricle, EL = energy loss, LVOT = left ventricle outflow tract, KE = kinetic energy, DCM = dilated cardiomyopathy, 4D flow CMR = 4D-flow cardiovascular magnetic resonance, LVSD: left ventricle systolic dysfunction, IVC = isovolumic contraction, HCM = hypertrophic cardiomyopathy, DM = diabetes mellitus, LVOT = left ventricle outflow tract, ELSYS: Systolic energy loss, ELED: End-diastolic energy loss, RFCA: radiofrequency catheter ablation, NIDCM = nonischemic dilated cardiomyopathy, HOCM = hypertrophic obstructive CMP, HNCM = hypertrophic nonobstructive CMP, LVEF: left ventricle ejection fraction, IVR: isovolumic relaxation, ESRD: end-stage renal disease, HT: hyperthyroidism, * DAS-RS1 (Hitachi-Aloka Medical Ltd., Tokyo, Japan) | | | | | |

| **Supplementary table 4.** Included 4D flow CMR studies | | | | | |
| --- | --- | --- | --- | --- | --- |
| Study, year | Software platform | Tenporal resolution (msec)* | Healthy controls (n) | Patient Group (n) | Notes/clinical implications |
| Arvidsson, 2017 [s.49] | Custom software & Segment† | 40 | 25 | Elite athletes (14) /DCM with LBBB (2) | Controls and athletes higher level of apicobasal forces. Patients with HF and LBBB had more horizontal distribution of forces. |
| Bolger, 2007 [s.41] | Custom software & EnSight‡ | 40 | 17 | DCM (1) | Higher Vdirect in controls compared with DCM. KE loss of Vdirect < KE loss of V retained in controls. This difference not present in DCM patients. |
| Calkoen, 2015 [s.50] | MASS§ | 30 | 30 | Surgically corrected AVSD(32) | Vdirect is smaller in corrected AVSD patients compared with controls. |
| Calkoen, 2015 [s.2] | MASS§ | 30 | 30 | Surgically corrected AVSD (32) | Absence of the diastolic vortex ring is more common in corrected AVSD patients compared to controls. Vortex rings in the patients had more anterior and apical position, more elliptical shape and oblique orientation. |
| Calkoen, 2015 [s.51] | MASS§ | 30 | 25 | Surgically corrected AVSD (25) | More laterally directed peak inflow flow vector in corrected AVSD patients compared with controls. |
| Crandon, 2018 [s.52] | MASS§ | 30 | 53 | - | KEE/KEA ratio demonstrated good correlation with the E/A ratio in healthy individuals. Peak E and Peak A KE demosntrated better correlation with age than Peak E and A values. |
| Elbaz, 2014 [s.53] | MASS§ | 30 | 24 | - | Distinct diastolic vortices during E and A waves, A-wave vortex being closer to mitral annulus, LV long axis and more elliptical in shape. |
| Elbaz, 2017 [s.54] | MASS§ | 30 | 30 | Surgically corrected AVSD (32) | Viscous EL was higher when vortex pattern was disturbed and highest when early diastolic vortex was absent. |
| Eriksson, 2011 [s.40] | Segment†, Ensight‡ | 40 | 13 | DCM (1) | Vdirect is the greatest portion of LV inflow; is closest to LVOT and angle of the mean flow vector of Vdirect to LVOT long axis is smallest among all volumes. |
| Eriksson, 2013 [s.55] | Segment†, Ensight‡ | 40 | 10 | DCM (10) | Vdirect is smaller in DCM patients compared with controls. |
| Eriksson, 2016 [s.56] | Segment†, Custom software | 40 | 10 | DCM(10) | Cited by [s.25]. Ratio of short-axis/long-axis forces was greater in HF patients compared with controls. |
| Foll, 2013 [s.1] | Ensight‡ | N/A | 24 | - | 2D parameters of vortex size and localization. Larger number of vortices with higher velocity distribution are found in younger patients. Vortex size at the basal segments were smaller in women. Vortex number, size and velocities correlated with LV end-diastolic volume, LV length and LVEF. |
| Garg, 2018 [s.25] | MASS | 30 | 20 | MI (48) | KE/LVEDV higher in controls compared with patients with ischemic LVSD/  Time to peak KEE difference between apex and base was greater in ischemic LVSD patients compared with controls. |
| Kamphuis, 2018 [s.14] | MASS | 30 | 12 | - | Time averaged EL over E and A waves shows better reproducibility compared with peak E and A EL values in healthy controls. |
| Kamphuis, 2018 [s.57] | MASS | 30 | 10 | - | Validation of particle tracing for determination of LV volume parameters (parameters of LV transport mechanics) |
| Kanski, 2015 [s.26] | Segment† with a custom module | 40 | 12 | HF (29) | Average systolic KE is higher and systolic KE/LVEDV is lower in patients compared with controls. Diastolic KE doesn’t differ between patients and controls. Smaller fraction of diastolic average KE inside the vortex ring is observed in patients vs controls. |
| Rutkowski, 2019 [s.21] | Ensight‡ | 14 | 15 | Single Ventricle + Fontan circulation (11) | No difference with regard to Reynolds number or vorticity between healthy controls and Fontan patients. Kinetic energy was higher and efficiency index (flow/KE) lower in patients with Fontan circulation. |
| Samnoy, 2017 [s.58] | Custom software | 15-17 | 13 | - | Systolic peak velocities were higher in basal segments compared to apical segments. The inclination angle of momentum vector moves has a small angle to LVOT long axis in end-systole. |
| Schafer, 2016 [s.20] | Matlab  Velomap  Paraview for 3D vorticity quantifiaction | 50 | 10 | PH patients (9 idiopathic and 4 scleroderma-associated) (13) | LV E-wave vorticity was significantly decreased in PH patients compared with controls and was associated with eccentricity index as well as LV and RV diastolic dysfunction. |
| Sjoberg, 2018 [s.37] | Segment† with a custom module | 40 | 14 | TOF (15) | Systolic KE parameters are significantly higher in controls compared with patients with repaired TOF |
| Steding-Ehrenborg, 2016 [s.38] | Segment† with a custom module | 40 | 14 | Elite athletes (14) | Peak early diastolic LV KE was lower in athletes compared to controls. |
| Stoll, 2018 [s.33] | CMR42\|, Segment†, Ensight‡ | N/A | 45 | - | Reference values for parameters of LV transport mechanics in healthy adults. |
| Svalbring, 2016 [s.39] | Ensight‡ | 40 | 10 | Chronic ischemic disease (26) | Vdirect correlated with LVEF and was higher in controls as well as patients with lower LVEDVI. |
| Van Ooij, 2016 [s.59] | Ensight‡, Mimics¶ | N/A | 10 | HCM (35) | Viscous EL in LVOT correlated with extracellular volume fraction by T1 mapping. |
| Zajac, 2015 [s.27] | Segment†, Ensight‡ Custom software | 40 | 11 | LVSD (9) | Peak A turbulent KE as well as total diastolic TKE was higher in DCM patients compared to controls. |
| N/A = not available, LV = left ventricle, LBBB = left bundle branch block, DCM = dilated cardiomyopathy, AVSD = atrioventricular septal defect, KEE/KEA = kinetic energy at E wave and A wave peaks, 2D-PC MRI: 2 dimensional phase contrast magnetic resonance imaging, LVOT = left ventricle outflow tract, LVSD = LV systolic dysfunction, EL = energy loss, MRI = magnetic resonance imaging, HF = heart failure, LVEDV = LV end diastolic volume, PH = pulmonary hypertension, TKE = turbulant kinetic energy. * Number of reconstructed phases, † Medviso AB, Lund, Sweden, ‡ CEI Inc, Apex, NC, US, § MASS, LUMC, Leiden, NL, ⁑ Kitware, Clifton, NY, US, \| Circle Cardiovascular Imaging Inc. Calcary, Canada, ¶ Materialise, Leuven, Belgium, ● included manually | | | | | |

| **Supplementary table 5.** Intraventricular velocity and pressure profiles | | | | | | |
| --- | --- | --- | --- | --- | --- | --- |
| **Parameter** | **Definition** | **EPIV** | **VFM** | **4D flow CMR** | **Clinical Implications** | **Ref.** |
| **Diastolic velocities** |  |  |  |  |  |  |
| *Atrioventricular velocity gradient* | Velocity gradient between mid-LV and left atrium | + | - | - | Decreased early diastolic atrioventricular and increased intraventricular velocity gradients with increasing degree of LVDD in patients with HCM. | [s.34] |
| *Intraventricular apicobasal velocity gradient* | Velocity gradient profile with sampling cursors placed on the center of the inflow at the baseline, middle and apex of LV | - | + | - | Increased intraventricular velocity gradient with increasing degree of LVDD. | [s.42] |
| *Pre-ejectional flow velocity in LVOT* (V_LVOT_) | Velocity tracing over a sampling line drawn from LV apex to aortic valve in the pre-ejection frame | - | + | - | Significantly lower in patients with increasing degrees of LVSD. Proposed as a surrogate marker of momentum transfer from diastole to systole. | [s.3] |
| **Systolic velocities** |  |  |  |  |  |  |
| *Flow force angle* | Angle of mean systolic momentum vector to LV transverse axis (2D) | + | - | - | Flow force angle was higher in healthy controls compared to patients with DCM. | [s.24] |
| *Direction of systolic momentum vector* | Angle of the systolic momentum vector to left ventricular outflow tract (3D) | - | - | + | Momentum vector of the ejecting components of the blood in LV displayed the minimal angle to the LVOT long axis. | [s.40] |
| **Pressure Gradients** |  |  |  |  |  |  |
| *Apicobasal pressure gradient* | Pressure gradients in LV apicobasal direction | + | + | + | Early diastolic IV gradient was decreased while mid and late diastolic IV gradient was increased in patients with thyrotoxic cardiomyopathy. | [s.7] |
| *Early diastolic pressure gradient in LVOT* | Pressure gradients with sampling cursors placed in mid LV and LVOT | + | - | - | Early diastolic pressure gradient in LVOT was significantly decreased in diastolic dysfunction grade 2 and 3. | [s.34] |
| **Intraventricular Hemodynamic Forces** |  |  |  |  |  |  |
| *Hemodynamic forces in horizontal and vertical axis* | Integral of LV pressure gradient multiplied by the total voxel volume over the entire LV. | - | - | + | Forces in the vertical direction predominate in LV, in contrast, vertical and horizontal forces are more even for RV. | [s.49] |
| *Short Axis/Long-axis hemodynamic force ratio* | Ratio of short-axis to long-axis hemodynamic forces | - | - | + | Short-axis/long-axis hemodynamic force ratios were lower in healthy controls compared with patients with DCM. | [s.56] |
| E-PIV: echocardiographic particle image velocimetry, VFM: vector flow mapping, 4D flow CMR: 4-dimensional flow cardiovascular magnetic resonance, LVDD: left ventricle diastolic dysfunction, HCM: hypertrophic cardiomyopathy, LVOT: left ventricle outflow tract, LVSD: left ventricle systolic dysfunction, RV: right ventricle, DCM: dilated cardiomyopathy, (-) not available, (+) available | | | | | | |

| 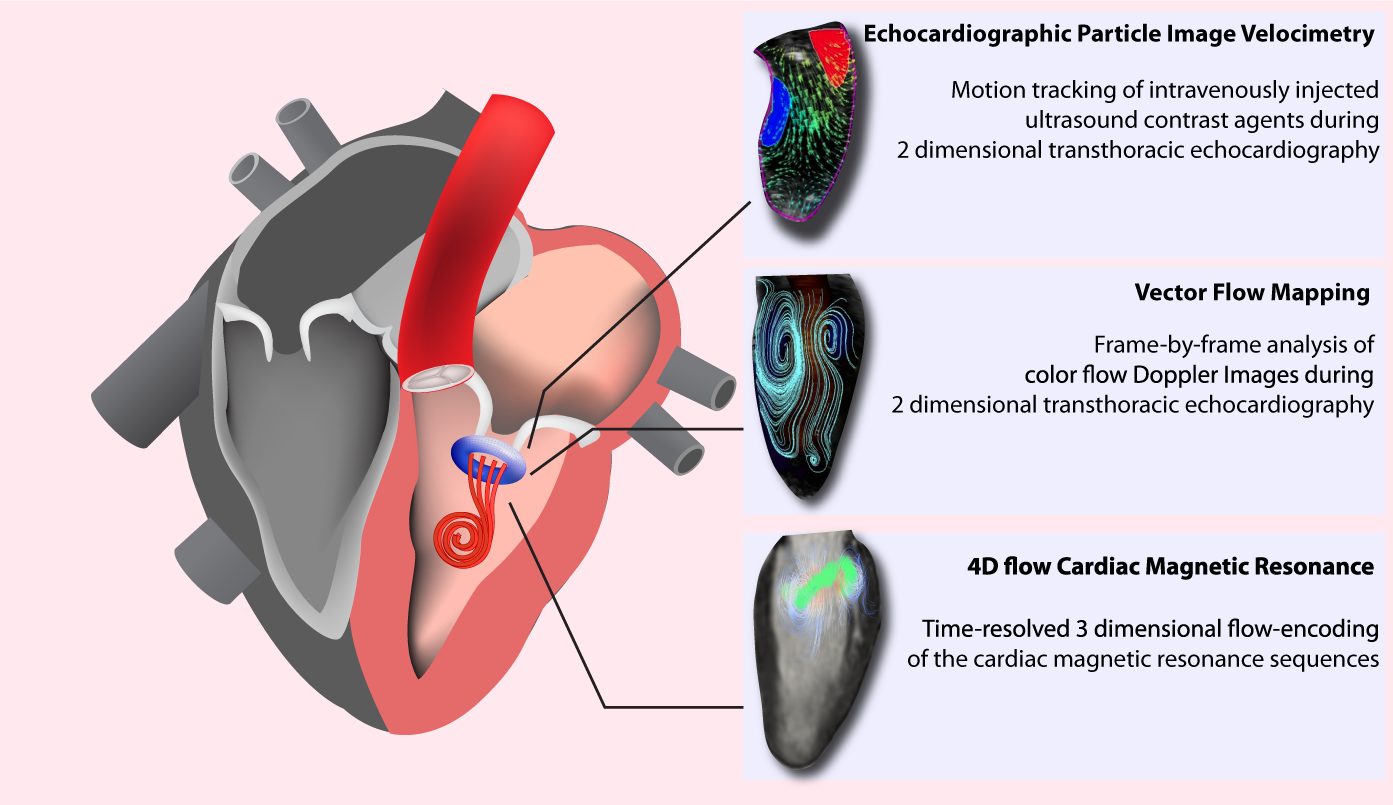 |
| --- |
| Online resource 6. Graphical abstract. Noninvasive flow visualization with multimodality imaging. |

**Online resource 7. Supplementary references**

s.1. Foll D, Taeger S, Bode C, Jung B, Markl M. Age, gender, blood pressure, and ventricular geometry influence normal 3D blood flow characteristics in the left heart. Eur Heart J Cardiovasc Imaging. 2013;14(4):366-73.

s.2. Calkoen EE, Elbaz MS, Westenberg JJ, Kroft LJ, Hazekamp MG, Roest AA, et al. Altered left ventricular vortex ring formation by 4-dimensional flow magnetic resonance imaging after repair of atrioventricular septal defects. J Thorac Cardiovasc Surg. 2015;150(5):1233-40 e1.

s.3. Li Q, Huang L, Ma N, Li Z, Han Y, Wu L, et al. Relationship between left ventricular vortex and preejectional flow velocity during isovolumic contraction studied by using vector flow mapping. Echocardiography. 2019;36(3):558-66.

s.4. Suwa K, Saitoh T, Takehara Y, Sano M, Saotome M, Urushida T, et al. Intra-left ventricular flow dynamics in patients with preserved and impaired left ventricular function: Analysis with 3D cine phase contrast MRI (4D-Flow). J Magn Reson Imaging. 2016;44(6):1493-503.

s.5. Yoshida S, Miyagawa S, Fukushima S, Yoshikawa Y, Hata H, Saito S, et al. Cardiac Function and Type of Mitral Valve Surgery Affect Postoperative Blood Flow Pattern in the Left Ventricle. Circ J. 2018;83(1):130-8.

s.6. Zhang H, Liu L, Chen L, Ma N, Zhou L, Liu Y, et al. The evolution of intraventricular vortex during ejection studied by using vector flow mapping. Echocardiography. 2013;30(1):27-36.

s.7. Zhou BY, Xie MX, Wang J, Wang XF, Lv Q, Liu MW, et al. Relationship between the abnormal diastolic vortex structure and impaired left ventricle filling in patients with hyperthyroidism. Medicine (Baltimore). 2017;96(17):e6711.

s.8. Bermejo J, Benito Y, Alhama M, Yotti R, Martinez-Legazpi P, Del Villar CP, et al. Intraventricular vortex properties in nonischemic dilated cardiomyopathy. Am J Physiol Heart Circ Physiol. 2014;306(5):H718-29.

s.9. Chen R, Zhao BW, Wang B, Tang HL, Li P, Pan M, et al. Assessment of left ventricular hemodynamics and function of patients with uremia by vortex formation using vector flow mapping. Echocardiography. 2012;29(9):1081-90.

s.10. Goliasch G, Goscinska-Bis K, Caracciolo G, Nakabo A, Smolka G, Pedrizzetti G, et al. CRT improves LV filling dynamics: insights from echocardiographic particle imaging velocimetry. JACC Cardiovasc Imaging. 2013;6(6):704-13.

s.11. Agati L, Cimino S, Tonti G, Cicogna F, Petronilli V, De Luca L, et al. Quantitative analysis of intraventricular blood flow dynamics by echocardiographic particle image velocimetry in patients with acute myocardial infarction at different stages of left ventricular dysfunction. Eur Heart J Cardiovasc Imaging. 2014;15(11):1203-12.

s.12. Abe H, Caracciolo G, Kheradvar A, Pedrizzetti G, Khandheria BK, Narula J, et al. Contrast echocardiography for assessing left ventricular vortex strength in heart failure: a prospective cohort study. Eur Heart J Cardiovasc Imaging. 2013;14(11):1049-60.

s.13. Cimino S, Pedrizzetti G, Tonti G, Canali E, Petronilli V, De Luca L, et al. In vivo analysis of intraventricular fluid dynamics in healthy hearts. Eur J Mech B Fluids. 2012;35:40-6.

s.14. Kamphuis VP, Westenberg JJM, van der Palen RLF, van den Boogaard PJ, van der Geest RJ, de Roos A, et al. Scan-rescan reproducibility of diastolic left ventricular kinetic energy, viscous energy loss and vorticity assessment using 4D flow MRI: analysis in healthy subjects. Int J Cardiovasc Imaging. 2018;34(6):905-20.

s.15. Kutty S, Li L, Danford DA, Houle H, Datta S, Mancina J, et al. Effects of right ventricular hemodynamic burden on intraventricular flow in tetralogy of fallot: an echocardiographic contrast particle imaging velocimetry study. J Am Soc Echocardiogr. 2014;27(12):1311-8.

s.16. Lampropoulos K, Budts W, Van de Bruaene A, Troost E, van Melle JP. Visualization of the intracavitary blood flow in systemic ventricles of Fontan patients by contrast echocardiography using particle image velocimetry. Cardiovasc Ultrasound. 2012;10(1):5.

s.17. Martinez-Legazpi P, Bermejo J, Benito Y, Yotti R, Perez Del Villar C, Gonzalez-Mansilla A, et al. Contribution of the diastolic vortex ring to left ventricular filling. J Am Coll Cardiol. 2014;64(16):1711-21.

s.18. Rodriguez Munoz D, Moya Mur JL, Fernandez-Golfin C, Becker Filho DC, Gonzalez Gomez A, Fernandez Santos S, et al. Left ventricular vortices as observed by vector flow mapping: main determinants and their relation to left ventricular filling. Echocardiography. 2015;32(1):96-105.

s.19. Ro R, Halpern D, Sahn DJ, Homel P, Arabadjian M, Lopresto C, et al. Vector flow mapping in obstructive hypertrophic cardiomyopathy to assess the relationship of early systolic left ventricular flow and the mitral valve. J Am Coll Cardiol. 2014;64(19):1984-95.

s.20. Schafer M, Browning J, Schroeder JD, Shandas R, Kheyfets VO, Buckner JK, et al. Vorticity is a marker of diastolic ventricular interdependency in pulmonary hypertension. Pulm Circ. 2016;6(1):46-54.

s.21. Rutkowski DR, Barton G, Francois CJ, Bartlett HL, Anagnostopoulos PV, Roldan-Alzate A. Analysis of cavopulmonary and cardiac flow characteristics in fontan Patients: Comparison with healthy volunteers. J Magn Reson Imaging. 2019;49(6):1786-99.

s.22. Gurel E, Prinz C, Van Casteren L, Gao H, Willems R, Voigt JU. The Impact of Function-Flow Interaction on Left Ventricular Efficiency in Patients with Conduction Abnormalities: A Particle Image Velocimetry and Tissue Doppler Study. J Am Soc Echocardiogr. 2016;29(5):431-40.

s.23. Hong GR, Pedrizzetti G, Tonti G, Li P, Wei Z, Kim JK, et al. Characterization and quantification of vortex flow in the human left ventricle by contrast echocardiography using vector particle image velocimetry. JACC Cardiovasc Imaging. 2008;1(6):705-17.

s.24. Tang C, Zhu Y, Zhang J, Niu C, Liu D, Liao Y, et al. Analysis of left ventricular fluid dynamics in dilated cardiomyopathy by echocardiographic particle image velocimetry. Echocardiography. 2018;35(1):56-63.

s.25. Garg P, Crandon S, Swoboda PP, Fent GJ, Foley JRJ, Chew PG, et al. Left ventricular blood flow kinetic energy after myocardial infarction - insights from 4D flow cardiovascular magnetic resonance. J Cardiovasc Magn Reson. 2018;20(1):61.

s.26. Kanski M, Arvidsson PM, Toger J, Borgquist R, Heiberg E, Carlsson M, et al. Left ventricular fluid kinetic energy time curves in heart failure from cardiovascular magnetic resonance 4D flow data. J Cardiovasc Magn Reson. 2015;17(1):111.

s.27. Zajac J, Eriksson J, Dyverfeldt P, Bolger AF, Ebbers T, Carlhall CJ. Turbulent kinetic energy in normal and myopathic left ventricles. J Magn Reson Imaging. 2015;41(4):1021-9.

s.28. Ji L, Hu W, Yong Y, Wu H, Zhou L, Xu D. Left ventricular energy loss and wall shear stress assessed by vector flow mapping in patients with hypertrophic cardiomyopathy. Int J Cardiovasc Imaging. 2018;34(9):1383-91.

s.29. Li CM, Bai WJ, Liu YT, Tang H, Rao L. Dissipative energy loss within the left ventricle detected by vector flow mapping in diabetic patients with controlled and uncontrolled blood glucose levels. Int J Cardiovasc Imaging. 2017;33(8):1151-8.

s.30. Lin M, Hao L, Cao Y, Xie F, Han W, Rong B, et al. Successful radiofrequency catheter ablation of atrial fibrillation is associated with improvement in left ventricular energy loss and mechanics abnormalities. Int J Cardiovasc Imaging. 2019;35(3):427-35.

s.31. Wang Y, Ma R, Ding G, Hou D, Li Z, Yin L, et al. Left Ventricular Energy Loss Assessed by Vector Flow Mapping in Patients with Prediabetes and Type 2 Diabetes Mellitus. Ultrasound Med Biol. 2016;42(8):1730-40.

s.32. Zhong Y, Liu Y, Wu T, Song H, Chen Z, Zhu W, et al. Assessment of Left Ventricular Dissipative Energy Loss by Vector Flow Mapping in Patients With End-Stage Renal Disease. J Ultrasound Med. 2016;35(5):965-73.

s.33. Stoll VM, Loudon M, Eriksson J, Bissell MM, Dyverfeldt P, Ebbers T, et al. Test-retest variability of left ventricular 4D flow cardiovascular magnetic resonance measurements in healthy subjects. J Cardiovasc Magn Reson. 2018;20(1):15.

s.34. Prinz C, Lehmann R, Brandao da Silva D, Jurczak B, Bitter T, Faber L, et al. Echocardiographic particle image velocimetry for the evaluation of diastolic function in hypertrophic nonobstructive cardiomyopathy. Echocardiography. 2014;31(7):886-94.

s.35. Xu L, Sun C, Zhu X, Liu W, Ta S, Zhao D, et al. Characterization of left ventricle energy loss in healthy adults using vector flow mapping: Preliminary results. Echocardiography. 2017;34(5):700-8.

s.36. Akiyama K, Maeda S, Matsuyama T, Kainuma A, Ishii M, Naito Y, et al. Vector flow mapping analysis of left ventricular energetic performance in healthy adult volunteers. BMC Cardiovasc Disord. 2017;17(1):21.

s.37. Sjoberg P, Bidhult S, Bock J, Heiberg E, Arheden H, Gustafsson R, et al. Disturbed left and right ventricular kinetic energy in patients with repaired tetralogy of Fallot: pathophysiological insights using 4D-flow MRI. Eur Radiol. 2018;28(10):4066-76.

s.38. Steding-Ehrenborg K, Arvidsson PM, Toger J, Rydberg M, Heiberg E, Carlsson M, et al. Determinants of kinetic energy of blood flow in the four-chambered heart in athletes and sedentary controls. Am J Physiol Heart Circ Physiol. 2016;310(1):H113-22.

s.39. Svalbring E, Fredriksson A, Eriksson J, Dyverfeldt P, Ebbers T, Bolger AF, et al. Altered Diastolic Flow Patterns and Kinetic Energy in Subtle Left Ventricular Remodeling and Dysfunction Detected by 4D Flow MRI. PLoS One. 2016;11(8):e0161391.

s.40. Eriksson J, Dyverfeldt P, Engvall J, Bolger AF, Ebbers T, Carlhall CJ. Quantification of presystolic blood flow organization and energetics in the human left ventricle. Am J Physiol Heart Circ Physiol. 2011;300(6):H2135-41.

s.41. Bolger AF, Heiberg E, Karlsson M, Wigstrom L, Engvall J, Sigfridsson A, et al. Transit of blood flow through the human left ventricle mapped by cardiovascular magnetic resonance. J Cardiovasc Magn Reson. 2007;9(5):741-7.

s.42. Chen M, Jin JM, Zhang Y, Gao Y, Liu SL. Assessment of left ventricular diastolic dysfunction based on the intraventricular velocity difference by vector flow mapping. J Ultrasound Med. 2013;32(12):2063-71.

s.43. Fukuda N, Itatani K, Kimura K, Ebihara A, Negishi K, Uno K, et al. Prolonged vortex formation during the ejection period in the left ventricle with low ejection fraction: a study by vector flow mapping. J Med Ultrason (2001). 2014;41(3):301-10.

s.44. Nogami Y, Ishizu T, Atsumi A, Yamamoto M, Kawamura R, Seo Y, et al. Abnormal early diastolic intraventricular flow 'kinetic energy index' assessed by vector flow mapping in patients with elevated filling pressure. Eur Heart J Cardiovasc Imaging. 2013;14(3):253-60.

s.45. Nogami Y, Ishizu T, Atsumi A, Yamamoto M, Nakamura A, Machino-Ohtsuka T, et al. Diastolic suction in heart failure: impact of left ventricular geometry, untwist, and flow mechanics. Life Sci. 2014;102(2):111-7.

s.46. Zhang H, Zhang J, Zhu X, Chen L, Liu L, Duan Y, et al. The left ventricular intracavitary vortex during the isovolumic contraction period as detected by vector flow mapping. Echocardiography. 2012;29(5):579-87.

s.47. Zhang H, Ren X, Song J, Cao X, Wang B, Liu Y, et al. Intraventricular Isovolumic Relaxation Flow Patterns Studied by Using Vector Flow Mapping. Echocardiography. 2016;33(6):902-9.

s.48. Zhou BY, Wang J, Xie MX, Liu MW, Lv Q. Left ventricular systolic intraventricular flow field assessment in hyperthyroidism patients using vector flow mapping. J Huazhong Univ Sci Technolog Med Sci. 2015;35(4):574-8.

s.49. Arvidsson PM, Toger J, Carlsson M, Steding-Ehrenborg K, Pedrizzetti G, Heiberg E, et al. Left and right ventricular hemodynamic forces in healthy volunteers and elite athletes assessed with 4D flow magnetic resonance imaging. Am J Physiol Heart Circ Physiol. 2017;312(2):H314-H28.

s.50. Calkoen EE, de Koning PJ, Blom NA, Kroft LJ, de Roos A, Wolterbeek R, et al. Disturbed Intracardiac Flow Organization After Atrioventricular Septal Defect Correction as Assessed With 4D Flow Magnetic Resonance Imaging and Quantitative Particle Tracing. Invest Radiol. 2015;50(12):850-7.

s.51. Calkoen EE, Roest AA, Kroft LJ, van der Geest RJ, Jongbloed MR, van den Boogaard PJ, et al. Characterization and improved quantification of left ventricular inflow using streamline visualization with 4DFlow MRI in healthy controls and patients after atrioventricular septal defect correction. J Magn Reson Imaging. 2015;41(6):1512-20.

s.52. Crandon S, Westenberg JJM, Swoboda PP, Fent GJ, Foley JRJ, Chew PG, et al. Impact of Age and Diastolic Function on Novel, 4D flow CMR Biomarkers of Left Ventricular Blood Flow Kinetic Energy. Sci Rep. 2018;8(1):14436.

s.53. Elbaz MS, Calkoen EE, Westenberg JJ, Lelieveldt BP, Roest AA, van der Geest RJ. Vortex flow during early and late left ventricular filling in normal subjects: quantitative characterization using retrospectively-gated 4D flow cardiovascular magnetic resonance and three-dimensional vortex core analysis. J Cardiovasc Magn Reson. 2014;16(1):78.

s.54. Elbaz MS, van der Geest RJ, Calkoen EE, de Roos A, Lelieveldt BP, Roest AA, et al. Assessment of viscous energy loss and the association with three-dimensional vortex ring formation in left ventricular inflow: In vivo evaluation using four-dimensional flow MRI. Magn Reson Med. 2017;77(2):794-805.

s.55. Eriksson J, Bolger AF, Ebbers T, Carlhall CJ. Four-dimensional blood flow-specific markers of LV dysfunction in dilated cardiomyopathy. Eur Heart J Cardiovasc Imaging. 2013;14(5):417-24.

s.56. Eriksson J, Bolger AF, Ebbers T, Carlhall CJ. Assessment of left ventricular hemodynamic forces in healthy subjects and patients with dilated cardiomyopathy using 4D flow MRI. Physiol Rep. 2016;4(3):e12685.

s.57. Kamphuis VP, van der Palen RLF, de Koning PJH, Elbaz MSM, van der Geest RJ, de Roos A, et al. In-scan and scan-rescan assessment of LV in- and outflow volumes by 4D flow MRI versus 2D planimetry. J Magn Reson Imaging. 2018;47(2):511-22.

s.58. Samnoy SF, Cuypers J, Greve G, Larsen TH. 4D left ventricular resultant wall motion and blood flow assessed by phase-shift velocity mapping at high-field 3T MRI. Clin Physiol Funct Imaging. 2017;37(6):615-21.

s.59. van Ooij P, Allen BD, Contaldi C, Garcia J, Collins J, Carr J, et al. 4D flow MRI and T1 -Mapping: Assessment of altered cardiac hemodynamics and extracellular volume fraction in hypertrophic cardiomyopathy. J Magn Reson Imaging. 2016;43(1):107-14.
