# Supplementary material for: Evaluation of intraventricular flow by multimodality imaging: a review and meta-analysis
Source: Cardiovasc Ultrasound. 2021 Dec 8;19:38. doi: 10.1186/s12947-021-00269-8 (PMC8653587; doi:10.1186/s12947-021-00269-8)
Supplement: Supplementary file 2 — Additional file 2. Supplementary references [file 12947_2021_269_MOESM2_ESM.docx]

**Additional file 2. Supplementary references**

s.1. Foll D, Taeger S, Bode C, Jung B, Markl M. Age, gender, blood pressure, and ventricular geometry influence normal 3D blood flow characteristics in the left heart. Eur Heart J Cardiovasc Imaging. 2013;14(4):366-73.

s.2. Calkoen EE, Elbaz MS, Westenberg JJ, Kroft LJ, Hazekamp MG, Roest AA, et al. Altered left ventricular vortex ring formation by 4-dimensional flow magnetic resonance imaging after repair of atrioventricular septal defects. J Thorac Cardiovasc Surg. 2015;150(5):1233-40 e1.

s.3. Li Q, Huang L, Ma N, Li Z, Han Y, Wu L, et al. Relationship between left ventricular vortex and preejectional flow velocity during isovolumic contraction studied by using vector flow mapping. Echocardiography. 2019;36(3):558-66.

s.4. Suwa K, Saitoh T, Takehara Y, Sano M, Saotome M, Urushida T, et al. Intra-left ventricular flow dynamics in patients with preserved and impaired left ventricular function: Analysis with 3D cine phase contrast MRI (4D-Flow). J Magn Reson Imaging. 2016;44(6):1493-503.

s.5. Yoshida S, Miyagawa S, Fukushima S, Yoshikawa Y, Hata H, Saito S, et al. Cardiac Function and Type of Mitral Valve Surgery Affect Postoperative Blood Flow Pattern in the Left Ventricle. Circ J. 2018;83(1):130-8.

s.6. Zhang H, Liu L, Chen L, Ma N, Zhou L, Liu Y, et al. The evolution of intraventricular vortex during ejection studied by using vector flow mapping. Echocardiography. 2013;30(1):27-36.

s.7. Zhou BY, Xie MX, Wang J, Wang XF, Lv Q, Liu MW, et al. Relationship between the abnormal diastolic vortex structure and impaired left ventricle filling in patients with hyperthyroidism. Medicine (Baltimore). 2017;96(17):e6711.

s.8. Bermejo J, Benito Y, Alhama M, Yotti R, Martinez-Legazpi P, Del Villar CP, et al. Intraventricular vortex properties in nonischemic dilated cardiomyopathy. Am J Physiol Heart Circ Physiol. 2014;306(5):H718-29.

s.9. Chen R, Zhao BW, Wang B, Tang HL, Li P, Pan M, et al. Assessment of left ventricular hemodynamics and function of patients with uremia by vortex formation using vector flow mapping. Echocardiography. 2012;29(9):1081-90.

s.10. Goliasch G, Goscinska-Bis K, Caracciolo G, Nakabo A, Smolka G, Pedrizzetti G, et al. CRT improves LV filling dynamics: insights from echocardiographic particle imaging velocimetry. JACC Cardiovasc Imaging. 2013;6(6):704-13.

s.11. Agati L, Cimino S, Tonti G, Cicogna F, Petronilli V, De Luca L, et al. Quantitative analysis of intraventricular blood flow dynamics by echocardiographic particle image velocimetry in patients with acute myocardial infarction at different stages of left ventricular dysfunction. Eur Heart J Cardiovasc Imaging. 2014;15(11):1203-12.

s.12. Abe H, Caracciolo G, Kheradvar A, Pedrizzetti G, Khandheria BK, Narula J, et al. Contrast echocardiography for assessing left ventricular vortex strength in heart failure: a prospective cohort study. Eur Heart J Cardiovasc Imaging. 2013;14(11):1049-60.

s.13. Cimino S, Pedrizzetti G, Tonti G, Canali E, Petronilli V, De Luca L, et al. In vivo analysis of intraventricular fluid dynamics in healthy hearts. Eur J Mech B Fluids. 2012;35:40-6.

s.14. Kamphuis VP, Westenberg JJM, van der Palen RLF, van den Boogaard PJ, van der Geest RJ, de Roos A, et al. Scan-rescan reproducibility of diastolic left ventricular kinetic energy, viscous energy loss and vorticity assessment using 4D flow MRI: analysis in healthy subjects. Int J Cardiovasc Imaging. 2018;34(6):905-20.

s.15. Kutty S, Li L, Danford DA, Houle H, Datta S, Mancina J, et al. Effects of right ventricular hemodynamic burden on intraventricular flow in tetralogy of fallot: an echocardiographic contrast particle imaging velocimetry study. J Am Soc Echocardiogr. 2014;27(12):1311-8.

s.16. Lampropoulos K, Budts W, Van de Bruaene A, Troost E, van Melle JP. Visualization of the intracavitary blood flow in systemic ventricles of Fontan patients by contrast echocardiography using particle image velocimetry. Cardiovasc Ultrasound. 2012;10(1):5.

s.17. Martinez-Legazpi P, Bermejo J, Benito Y, Yotti R, Perez Del Villar C, Gonzalez-Mansilla A, et al. Contribution of the diastolic vortex ring to left ventricular filling. J Am Coll Cardiol. 2014;64(16):1711-21.

s.18. Rodriguez Munoz D, Moya Mur JL, Fernandez-Golfin C, Becker Filho DC, Gonzalez Gomez A, Fernandez Santos S, et al. Left ventricular vortices as observed by vector flow mapping: main determinants and their relation to left ventricular filling. Echocardiography. 2015;32(1):96-105.

s.19. Ro R, Halpern D, Sahn DJ, Homel P, Arabadjian M, Lopresto C, et al. Vector flow mapping in obstructive hypertrophic cardiomyopathy to assess the relationship of early systolic left ventricular flow and the mitral valve. J Am Coll Cardiol. 2014;64(19):1984-95.

s.20. Schafer M, Browning J, Schroeder JD, Shandas R, Kheyfets VO, Buckner JK, et al. Vorticity is a marker of diastolic ventricular interdependency in pulmonary hypertension. Pulm Circ. 2016;6(1):46-54.

s.21. Rutkowski DR, Barton G, Francois CJ, Bartlett HL, Anagnostopoulos PV, Roldan-Alzate A. Analysis of cavopulmonary and cardiac flow characteristics in fontan Patients: Comparison with healthy volunteers. J Magn Reson Imaging. 2019;49(6):1786-99.

s.22. Gurel E, Prinz C, Van Casteren L, Gao H, Willems R, Voigt JU. The Impact of Function-Flow Interaction on Left Ventricular Efficiency in Patients with Conduction Abnormalities: A Particle Image Velocimetry and Tissue Doppler Study. J Am Soc Echocardiogr. 2016;29(5):431-40.

s.23. Hong GR, Pedrizzetti G, Tonti G, Li P, Wei Z, Kim JK, et al. Characterization and quantification of vortex flow in the human left ventricle by contrast echocardiography using vector particle image velocimetry. JACC Cardiovasc Imaging. 2008;1(6):705-17.

s.24. Tang C, Zhu Y, Zhang J, Niu C, Liu D, Liao Y, et al. Analysis of left ventricular fluid dynamics in dilated cardiomyopathy by echocardiographic particle image velocimetry. Echocardiography. 2018;35(1):56-63.

s.25. Garg P, Crandon S, Swoboda PP, Fent GJ, Foley JRJ, Chew PG, et al. Left ventricular blood flow kinetic energy after myocardial infarction - insights from 4D flow cardiovascular magnetic resonance. J Cardiovasc Magn Reson. 2018;20(1):61.

s.26. Kanski M, Arvidsson PM, Toger J, Borgquist R, Heiberg E, Carlsson M, et al. Left ventricular fluid kinetic energy time curves in heart failure from cardiovascular magnetic resonance 4D flow data. J Cardiovasc Magn Reson. 2015;17(1):111.

s.27. Zajac J, Eriksson J, Dyverfeldt P, Bolger AF, Ebbers T, Carlhall CJ. Turbulent kinetic energy in normal and myopathic left ventricles. J Magn Reson Imaging. 2015;41(4):1021-9.

s.28. Ji L, Hu W, Yong Y, Wu H, Zhou L, Xu D. Left ventricular energy loss and wall shear stress assessed by vector flow mapping in patients with hypertrophic cardiomyopathy. Int J Cardiovasc Imaging. 2018;34(9):1383-91.

s.29. Li CM, Bai WJ, Liu YT, Tang H, Rao L. Dissipative energy loss within the left ventricle detected by vector flow mapping in diabetic patients with controlled and uncontrolled blood glucose levels. Int J Cardiovasc Imaging. 2017;33(8):1151-8.

s.30. Lin M, Hao L, Cao Y, Xie F, Han W, Rong B, et al. Successful radiofrequency catheter ablation of atrial fibrillation is associated with improvement in left ventricular energy loss and mechanics abnormalities. Int J Cardiovasc Imaging. 2019;35(3):427-35.

s.31. Wang Y, Ma R, Ding G, Hou D, Li Z, Yin L, et al. Left Ventricular Energy Loss Assessed by Vector Flow Mapping in Patients with Prediabetes and Type 2 Diabetes Mellitus. Ultrasound Med Biol. 2016;42(8):1730-40.

s.32. Zhong Y, Liu Y, Wu T, Song H, Chen Z, Zhu W, et al. Assessment of Left Ventricular Dissipative Energy Loss by Vector Flow Mapping in Patients With End-Stage Renal Disease. J Ultrasound Med. 2016;35(5):965-73.

s.33. Stoll VM, Loudon M, Eriksson J, Bissell MM, Dyverfeldt P, Ebbers T, et al. Test-retest variability of left ventricular 4D flow cardiovascular magnetic resonance measurements in healthy subjects. J Cardiovasc Magn Reson. 2018;20(1):15.

s.34. Prinz C, Lehmann R, Brandao da Silva D, Jurczak B, Bitter T, Faber L, et al. Echocardiographic particle image velocimetry for the evaluation of diastolic function in hypertrophic nonobstructive cardiomyopathy. Echocardiography. 2014;31(7):886-94.

s.35. Xu L, Sun C, Zhu X, Liu W, Ta S, Zhao D, et al. Characterization of left ventricle energy loss in healthy adults using vector flow mapping: Preliminary results. Echocardiography. 2017;34(5):700-8.

s.36. Akiyama K, Maeda S, Matsuyama T, Kainuma A, Ishii M, Naito Y, et al. Vector flow mapping analysis of left ventricular energetic performance in healthy adult volunteers. BMC Cardiovasc Disord. 2017;17(1):21.

s.37. Sjoberg P, Bidhult S, Bock J, Heiberg E, Arheden H, Gustafsson R, et al. Disturbed left and right ventricular kinetic energy in patients with repaired tetralogy of Fallot: pathophysiological insights using 4D-flow MRI. Eur Radiol. 2018;28(10):4066-76.

s.38. Steding-Ehrenborg K, Arvidsson PM, Toger J, Rydberg M, Heiberg E, Carlsson M, et al. Determinants of kinetic energy of blood flow in the four-chambered heart in athletes and sedentary controls. Am J Physiol Heart Circ Physiol. 2016;310(1):H113-22.

s.39. Svalbring E, Fredriksson A, Eriksson J, Dyverfeldt P, Ebbers T, Bolger AF, et al. Altered Diastolic Flow Patterns and Kinetic Energy in Subtle Left Ventricular Remodeling and Dysfunction Detected by 4D Flow MRI. PLoS One. 2016;11(8):e0161391.

s.40. Eriksson J, Dyverfeldt P, Engvall J, Bolger AF, Ebbers T, Carlhall CJ. Quantification of presystolic blood flow organization and energetics in the human left ventricle. Am J Physiol Heart Circ Physiol. 2011;300(6):H2135-41.

s.41. Bolger AF, Heiberg E, Karlsson M, Wigstrom L, Engvall J, Sigfridsson A, et al. Transit of blood flow through the human left ventricle mapped by cardiovascular magnetic resonance. J Cardiovasc Magn Reson. 2007;9(5):741-7.

s.42. Chen M, Jin JM, Zhang Y, Gao Y, Liu SL. Assessment of left ventricular diastolic dysfunction based on the intraventricular velocity difference by vector flow mapping. J Ultrasound Med. 2013;32(12):2063-71.

s.43. Fukuda N, Itatani K, Kimura K, Ebihara A, Negishi K, Uno K, et al. Prolonged vortex formation during the ejection period in the left ventricle with low ejection fraction: a study by vector flow mapping. J Med Ultrason (2001). 2014;41(3):301-10.

s.44. Nogami Y, Ishizu T, Atsumi A, Yamamoto M, Kawamura R, Seo Y, et al. Abnormal early diastolic intraventricular flow 'kinetic energy index' assessed by vector flow mapping in patients with elevated filling pressure. Eur Heart J Cardiovasc Imaging. 2013;14(3):253-60.

s.45. Nogami Y, Ishizu T, Atsumi A, Yamamoto M, Nakamura A, Machino-Ohtsuka T, et al. Diastolic suction in heart failure: impact of left ventricular geometry, untwist, and flow mechanics. Life Sci. 2014;102(2):111-7.

s.46. Zhang H, Zhang J, Zhu X, Chen L, Liu L, Duan Y, et al. The left ventricular intracavitary vortex during the isovolumic contraction period as detected by vector flow mapping. Echocardiography. 2012;29(5):579-87.

s.47. Zhang H, Ren X, Song J, Cao X, Wang B, Liu Y, et al. Intraventricular Isovolumic Relaxation Flow Patterns Studied by Using Vector Flow Mapping. Echocardiography. 2016;33(6):902-9.

s.48. Zhou BY, Wang J, Xie MX, Liu MW, Lv Q. Left ventricular systolic intraventricular flow field assessment in hyperthyroidism patients using vector flow mapping. J Huazhong Univ Sci Technolog Med Sci. 2015;35(4):574-8.

s.49. Arvidsson PM, Toger J, Carlsson M, Steding-Ehrenborg K, Pedrizzetti G, Heiberg E, et al. Left and right ventricular hemodynamic forces in healthy volunteers and elite athletes assessed with 4D flow magnetic resonance imaging. Am J Physiol Heart Circ Physiol. 2017;312(2):H314-H28.

s.50. Calkoen EE, de Koning PJ, Blom NA, Kroft LJ, de Roos A, Wolterbeek R, et al. Disturbed Intracardiac Flow Organization After Atrioventricular Septal Defect Correction as Assessed With 4D Flow Magnetic Resonance Imaging and Quantitative Particle Tracing. Invest Radiol. 2015;50(12):850-7.

s.51. Calkoen EE, Roest AA, Kroft LJ, van der Geest RJ, Jongbloed MR, van den Boogaard PJ, et al. Characterization and improved quantification of left ventricular inflow using streamline visualization with 4DFlow MRI in healthy controls and patients after atrioventricular septal defect correction. J Magn Reson Imaging. 2015;41(6):1512-20.

s.52. Crandon S, Westenberg JJM, Swoboda PP, Fent GJ, Foley JRJ, Chew PG, et al. Impact of Age and Diastolic Function on Novel, 4D flow CMR Biomarkers of Left Ventricular Blood Flow Kinetic Energy. Sci Rep. 2018;8(1):14436.

s.53. Elbaz MS, Calkoen EE, Westenberg JJ, Lelieveldt BP, Roest AA, van der Geest RJ. Vortex flow during early and late left ventricular filling in normal subjects: quantitative characterization using retrospectively-gated 4D flow cardiovascular magnetic resonance and three-dimensional vortex core analysis. J Cardiovasc Magn Reson. 2014;16(1):78.

s.54. Elbaz MS, van der Geest RJ, Calkoen EE, de Roos A, Lelieveldt BP, Roest AA, et al. Assessment of viscous energy loss and the association with three-dimensional vortex ring formation in left ventricular inflow: In vivo evaluation using four-dimensional flow MRI. Magn Reson Med. 2017;77(2):794-805.

s.55. Eriksson J, Bolger AF, Ebbers T, Carlhall CJ. Four-dimensional blood flow-specific markers of LV dysfunction in dilated cardiomyopathy. Eur Heart J Cardiovasc Imaging. 2013;14(5):417-24.

s.56. Eriksson J, Bolger AF, Ebbers T, Carlhall CJ. Assessment of left ventricular hemodynamic forces in healthy subjects and patients with dilated cardiomyopathy using 4D flow MRI. Physiol Rep. 2016;4(3):e12685.

s.57. Kamphuis VP, van der Palen RLF, de Koning PJH, Elbaz MSM, van der Geest RJ, de Roos A, et al. In-scan and scan-rescan assessment of LV in- and outflow volumes by 4D flow MRI versus 2D planimetry. J Magn Reson Imaging. 2018;47(2):511-22.

s.58. Samnoy SF, Cuypers J, Greve G, Larsen TH. 4D left ventricular resultant wall motion and blood flow assessed by phase-shift velocity mapping at high-field 3T MRI. Clin Physiol Funct Imaging. 2017;37(6):615-21.

s.59. van Ooij P, Allen BD, Contaldi C, Garcia J, Collins J, Carr J, et al. 4D flow MRI and T1 -Mapping: Assessment of altered cardiac hemodynamics and extracellular volume fraction in hypertrophic cardiomyopathy. J Magn Reson Imaging. 2016;43(1):107-14.
