# Supplementary material for: Evaluation of intraventricular flow by multimodality imaging: a review and meta-analysis
Source: Cardiovasc Ultrasound. 2021 Dec 8;19:38. doi: 10.1186/s12947-021-00269-8 (PMC8653587; doi:10.1186/s12947-021-00269-8)
Supplement: Supplementary file 3 — Additional file 3. Graphical abstract. Noninvasive flow visualization with multimodality imaging. [file 12947_2021_269_MOESM3_ESM.pdf]

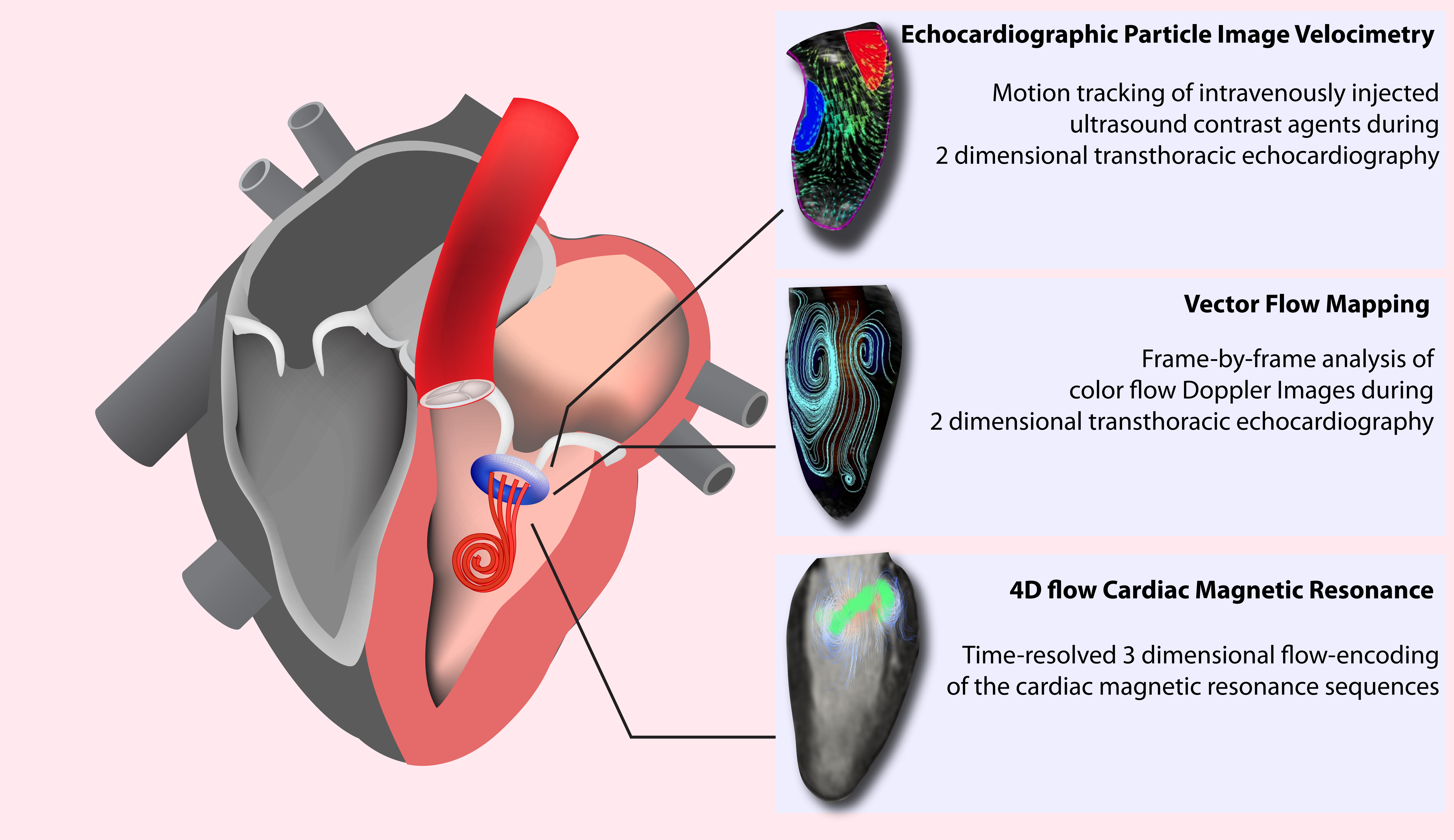
Echocardiographic Particle Image Velocimetry
Motion tracking of intravenously injected ultrasound contrast agents during 2 dimensional transthoracic echocardiography
Vector Flow Mapping
Frame-by-frame analysis of color flow Doppler Images during 2 dimensional transthoracic echocardiography
4D flow Cardiac Magnetic Resonance
Time-resolved 3 dimensional flow-encoding of the cardiac magnetic resonance sequences
